# Effects of Adding Tiotropium or Aclidinium as Triple Therapy Using Impulse Oscillometry in COPD

**DOI:** 10.1007/s00408-015-9839-y

**Published:** 2016-01-13

**Authors:** Arvind Manoharan, Ashley E. Morrison, Brian J. Lipworth

**Affiliations:** Scottish Centre for Respiratory Research, Ninewells Hospital and Medical School, University of Dundee, Dundee, DD1 9SY Scotland, UK

**Keywords:** COPD, Spirometry, Impulse oscillometry, Tiotropium, Aclidinium

## Abstract

**Introduction:**

Long-acting muscarinic antagonists confer improvements in spirometry when used in addition to inhaled corticosteroids and long-acting beta-agonists (ICS/LABA) in COPD. The dual objectives of this proof of concept study were to evaluate trough effects of tiotropium (TIO) or aclidinium (ACL) when used as triple therapy and to assess if impulse oscillometry (IOS) might be more sensitive than spirometry in detecting subtle differences in bronchodilator response.

**Methods:**

Patients with moderate to severe COPD already taking ICS/LABA were randomized to receive add-on therapy in cross-over fashion with either TIO 18 µg od or ACL 322 µg bid for 2–3 weeks each. Measurements of IOS, spirometry, 6-min walk test, St George’s Respiratory Questionnaire (SGRQ) and Baseline/Transition Dyspnoea Index (TDI) were made at baseline and after chronic dosing at trough (12 h for ACL and 24 h for TIO), in addition to domiciliary diurnal spirometry.

**Results:**

13 patients were completed: mean age 69 years, FEV_1_ 52 % predicted, FEV_1_/FVC 0.48, and R5 202 % predicted. There were no differences in any visit-based trough IOS or spirometry outcomes comparing TIO versus ACL. Resonant frequency but not total airway resistance at 5 Hz (R5) significantly improved from baseline with both treatments while peripheral airway resistance (R5–R20) significantly improved with ACL. Visit-based FEV_1_, and forced and relaxed vital capacity were also significantly improved from baseline with both treatments. There were no significant differences in diurnal FEV_1_ and FEV_6_ profiles between treatments. 6-min walk distance and post-walk fatigue significantly improved from baseline with ACL, while post-walk dyspnea improved with TIO. SGRQ symptom score significantly improved to a similar degree with both treatments. TDI significantly improved with ACL versus TIO by 1.54 units.

**Conclusion:**

We observed comparable bronchodilator efficacy at trough with TIO and ACL when used as triple therapy in COPD, while IOS was no more sensitive than spirometry.

## Introduction

Long-acting muscarinic antagonists (LAMA) are recommended in chronic obstructive pulmonary disease management guidelines [1] as bronchodilator therapy either alone or in combination with long-acting beta-agonists (LABA) and inhaled corticosteroids (ICS). Currently available LAMA include once daily tiotropium (TIO), glycopyrronium (GLYC) and umeclidinium or twice daily aclidinium (ACL). ACL and TIO have similar binding affinities for the M3 receptor and comparable kinetic selectivity for M3 over M2 receptors, while the duration of action for TIO is approximately two-fold longer than that for ACL [2]. For patients with more severe COPD the use of triple therapy with ICS/LABA/LAMA is advocated to improve outcomes including pulmonary function, quality of life, and exacerbations [3–9].

Few studies have however compared different LAMA when used as triple therapy. Once daily use of TIO or glycopyrronium versus placebo for 12 weeks as add-on therapy to ICS/LABA conferred similar improvements in spirometry and quality of life in patients with moderate to severe COPD [10]. In a chronic dosing comparison of once daily TIO and twice daily ACL as monotherapy for 2 weeks, the diurnal bronchodilator profile showed a noticeable decline in forced expiratory volume in 1 s (FEV_1_) between 12 and 24 h with TIO along with an improvement with ACL after the second evening dose over the same time period, such that the difference was significant [11]. In another study comparing TIO and ACL as monotherapy for 6 weeks, there were similar significant improvements in the 24 h FEV_1_ profile with both drugs compared with placebo, while only ACL significantly reduced early morning cough, wheeze, dyspnea, phlegm, and nighttime symptoms versus placebo [12]. In both of these studies [11, 12] there was no significant difference in morning pre dose trough FEV_1_ when comparing ACL and TIO after chronic dosing.

Spirometry involves a forced expiratory maneuver which may not be the ideal test to detect subtle improvements in airway caliber in COPD due to effort-dependent small airway closure. Impulse oscillometry (IOS) is an effort-independent test performed during normal quiet breathing, thereby obviating expiratory small airway closure [13]. IOS is easier to perform for patients with COPD during tidal breathing and measures the frequency dependence of airway resistance (R) and reactance (X). As previously described [13], IOS can be used to derive total airway resistance at 5 Hz (R5), central airway resistance at 20 Hz (R20), peripheral resistance (R5–R20), reactance at 5 Hz (X5), and area under the reactance curve (AX) as well as the resonant frequency (RF). In one study using IOS comparing TIO and placebo as add-on to ICS/LABA, there was no significant additive improvement on IOS outcomes with chronic dosing, despite a significant improvement in FEV_1_ [9]. However, we are not aware of any studies which have compared different LAMA as triple therapy using IOS.

The dual objectives of this proof of concept study were to evaluate the effects of TIO or ACL at trough when used as add-on therapy to pre-existing ICS/LABA and also to assess whether impulse oscillometry (IOS) might be more sensitive than spirometry in detecting subtle differences in bronchodilator efficacy. We also used domiciliary spirometry measurements to follow diurnal changes in airway caliber at steady state during each randomized treatment period.

## Methods

### Study Participants

Inclusion criteria were male or female volunteers aged 40–80 years with moderate to severe COPD on ICS/LABA, FEV_1_ 30–80 % and smoking history ≥10 pack-years. Exclusion criteria were other significant respiratory diseases; a COPD exacerbation or respiratory tract infection requiring systemic steroids, and/or antibiotics within 1 month (3 months if hospitalization was required) of the study commencement. The East of Scotland Research Ethics Service granted ethical approval (Ref: 13/ES/0122), and all patients provided written informed consent.

### Study Design

**Fig. 1:**
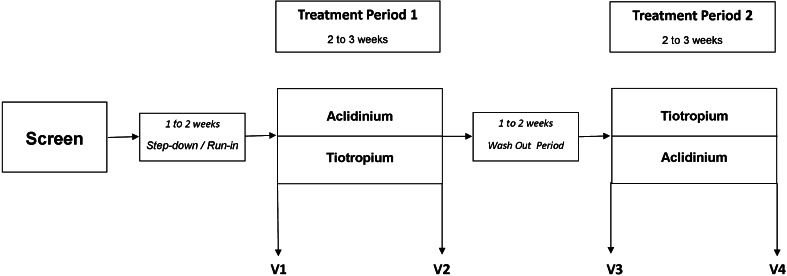
After a 1- to 2-week run-in, patients received either tiotropium 18 µg od or aclidinium 322 µg bid for 2–3 weeks each with a 1- to 2-week wash-out in between. Baseline values were measured at Visit 1/3 and after chronic dosing at Visit 2/4

We carried out a randomized, open-label, cross-over study (Fig. 1). Previously prescribed LAMA were stopped at the screening visit. After a 1- to 2-week period on ICS/LABA, patients were randomized to either ACL (Eklira Genuair, Astra Zeneca, Luton, UK) 322 µg bid or TIO (Spiriva HandiHaler, Boehringer, Bracknell, UK) 18 µg bid. Following the run-in period, at visit 1, baseline measurements were recorded. After 2–3 weeks on the study inhaler, participants returned to the department for visit 2. All measurements were taken at trough (i.e., 12 h post dose for ACL and ICS/LABA and 24 h post dose for TIO). Participants attended the department the same time during each study visit. After the first treatment period, participants entered a wash-out period of 1–2 weeks. The same process was repeated with the other study inhaler after cross-over.

### Primary and Secondary Outcomes

The primary outcome was change in trough R5 from baseline after chronic dosing. Secondary outcomes included change from baseline in the remaining IOS variables

(R20, R5–R20, X5, RF and AX), spirometry including FEV_1_, forced mid-expiratory flow between 25 and 75 % of forced vital capacity (FEF_25–75_), forced vital capacity (FVC), relaxed vital capacity (RVC), 6-min walk test (6MWT), domiciliary PiKo-6 measurements for FEV_1_ and FEV_6_, St George’s Respiratory Questionnaire (SGRQ) and Baseline/Transition Dyspnoea Index (BDI-TDI).

### Measurements

Impulse oscillometry (Masterscreen IOS, Höchberg, Germany) was performed in triplicate according to the manufacturer’s instructions. Spirometry was performed using a SuperSpiro (Micro Medical Ltd, Chatham, Kent, UK). Domiciliary FEV_1_ & FEV_6_ measurements were recorded using a handheld PiKo-6 monitor (n-Spire Health, Longmont, CO, USA). Domiciliary PiKo-6 measurements were recorded at trough for both drugs; and after each morning and evening dose of ACL (i.e., 2 h post dose); or at corresponding times after each morning dose of TIO (i.e., 2 and 14 h post dose).

### Statistical Analysis

The study was powered at 80 % to detect a 0.1 kPa L^−1^ s difference in the primary outcome of trough R5, assuming a within subject standard deviation of 0.13 kPa L^−1^ s, and an alpha error of 0.05 (two-tailed). Data were first examined for normality of distribution. Paired Students *t*-tests were used to compare between treatment effects at either baseline and after each chronic dosing, as well as within treatment effects comparing baseline versus chronic dosing. Repeated measures analysis of variance was used to assess the diurnal profile from serial domiciliary FEV_1_ and FEV_6_ measurements using the average from the last week of each randomized treatment period and the last week of each baseline.

## Results

**Fig. 2:**
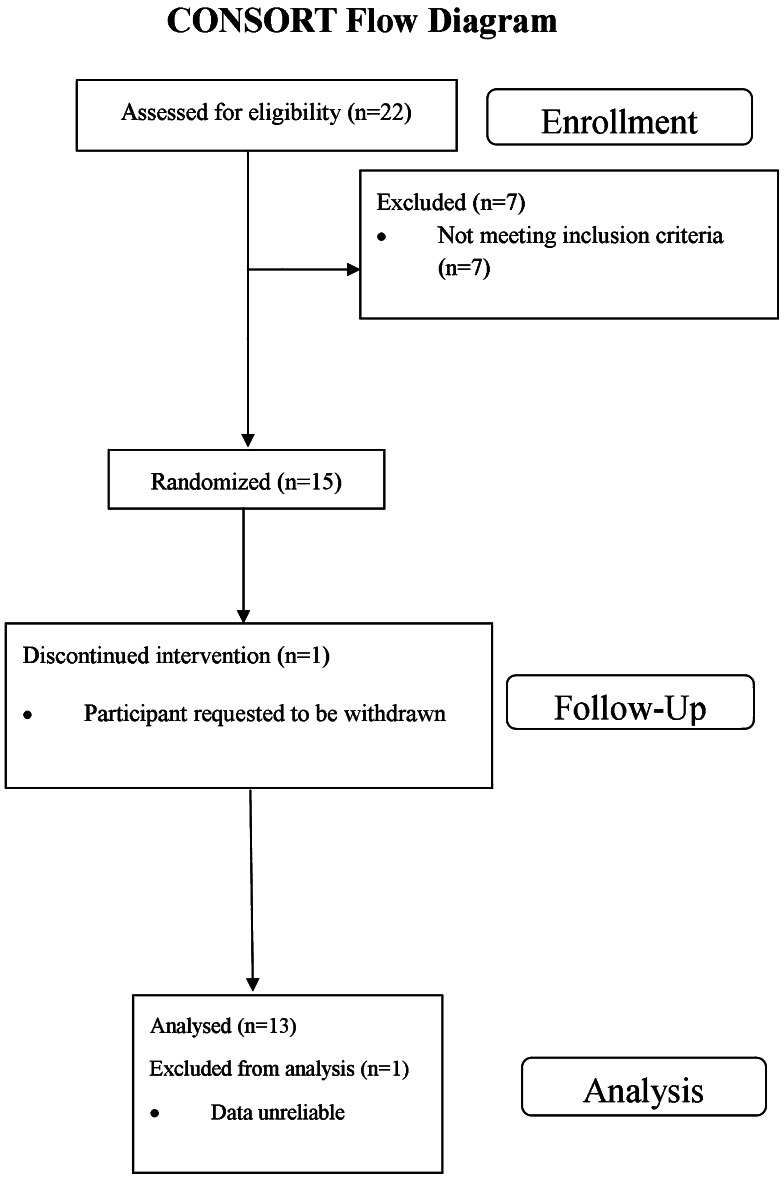
CONSORT diagram showing the flow of participants through the study

Thirteen patients were completed per protocol (Fig. 2): age 69 years, 10 males, and mean of 47 pack-years. Post-bronchodilator FEV_1_ was 52 % predicted with 10 % reversibility, FEV_1_/FVC ratio was 0.48, and R5 % was 202 % predicted. Nine patients were taking fluticasone/salmeterol, 3 budesonide/formoterol, and 1 beclomethasone/formoterol, with a mean beclomethasone equivalent dose of 1000 µg day^−1^. 11 patients were taking LAMA: 8 with TIO, 2 with ACL, and 1 with glycopyrronium.

**Fig. 3:**
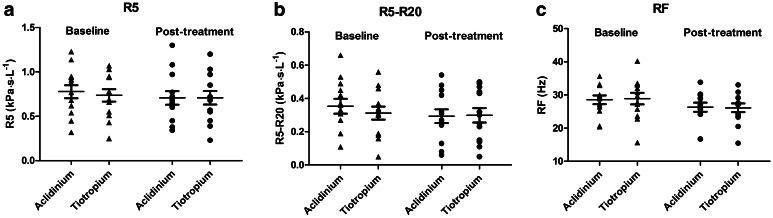
Effects on impulse oscillometry outcomes at baseline and post-treatment with either tiotropium or aclidinium. Data are depicted for individuals as well as means and SEM. **a** *R5* total airway resistance **b** *R5–R20* peripheral airway resistance **c** *RF* resonant frequency. There were significant improvements from baseline in RF with aclidinium (*P* < 0.05) and tiotropium (*P* < 0.01), and in R5–20 with tiotropium (*P* < 0.05). There were no significant differences between tiotropium and aclidinium in any oscillometry outcomes

**Fig. 4:**
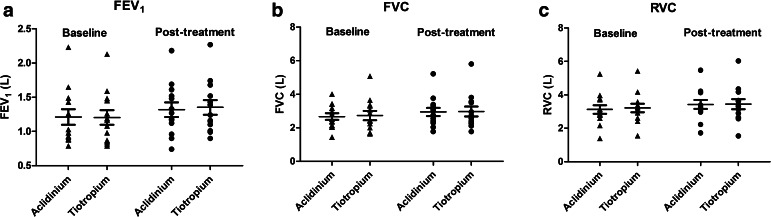
Effects on spirometry outcomes at baseline and post-treatment with either tiotropium or aclidinium. Data are depicted for individuals as well as means and SEM. **a** *FEV*_*1*_ forced expiratory volume in 1 s **b** *FVC* forced vital capacity **c** *RVC* relaxed vital capacity. There were significant improvements from baseline for FEV_1_ within aclidinium (*P* < 0.01) and tiotropium (*P* < 0.0001), for FVC within aclidinium (*P* < 0.05) and tiotropium (*P* < 0.01), and for RVC within aclidinium (*P* < 0.0001) and tiotropium (*P* < 0.05). There were no significant differences between tiotropium and aclidinium in any spirometry outcomes

Baseline values prior to randomized treatments were not significantly different, and there were also no significant differences in baselines according to visit sequence. There were no differences in any visit-based trough IOS or spirometry outcomes comparing TIO versus ACL (Table 1; Fig. 3). Resonant frequency (RF) but not total airway resistance at 5 Hz (R5) significantly improved from baseline within both treatments, while peripheral airway resistance (R5–R20) significantly improved with ACL (Tables 2, 3). Visit-based FEV_1_, FVC, and RVC were also significantly improved from baseline within both treatments (Tables 2, 3). There were no significant differences between treatments at any time points during the diurnal FEV_1_ and FEV_6_ profiles (Fig. 4).

**Table 1:** Change from baseline for aclidinium versus tiotropium

Parameter	Aclidinium	Tiotropium	Difference (95 % CI)	*P* value
FEV_1_(L)	0.11	0.15	−0.04 (−0.13, 0.05)	0.36
FVC (L)	0.28	0.24	0.03 (−0.16, 0.23)	0.72
FEF_25–75_ (L s^−1^)	0.02	0.06	−0.04 (−0.10, 0.01)	0.13
RVC (L)	0.30	0.22	0.08 (−0.12, 0.28)	0.39
RVC/FVC	0.01	−0.05	0.06 (−0.09, 0.21)	0.38
R5 (kPa L^−1^ s)	−0.07	−0.03	−0.04 (−0.12, 0.04)	0.29
R20 (kPa L^−1^ s)	−0.01	−0.02	0.01 (−0.05, 0.06)	0.80
R5–R20 (kPa L^−1^ s)	−0.06	−0.01	−0.05 (−0.11, 0.02)	0.13
X5 (kPa L^−1^ s)	0.03	0.05	−0.02 (−0.09, 0.06)	0.62
RF (Hz)	−2.22	−2.77	0.54 (−2.90, 3.99)	0.74
AX (kPa L^−1^)	−0.70	−0.55	−0.15 (−0.84, 0.54)	0.65
6MWT				
Distance (m)	36	9	27 (−2, 56)	0.07
Post-walk oxygen saturation (%)	0	0	0 (−2, 2)	0.93
Post-walk heart rate (bpm)	3	1	2 (−2, 6)	0.36
Post-walk dyspnoea (Borg scale)	−0.6	−0.7	0.1 (−0.6, 0.7)	0.79
Post-walk fatigue (Borg scale)	−0.5	−0.3	−0.2 (−0.6, 0.2)	0.34
SGRQ				
Symptoms score	−7.35	−7.31	−0.03 (−5.66, 5.59)	0.99
Total score	−0.97	−2.35	1.38 (−1.85, 4.61)	0.37

Mean values for change from baseline are shown each drug as well as the difference between the drugs

*FEV*_*1*_ forced expiratory volume in 1 s, *FVC* forced vital capacity, *FEF*_*25–75*_ forced mid-expiratory flow between 25 and 75 % of forced vital capacity, *RVC* relaxed vital capacity, *R5* total airway resistance at 5 Hz, *R20* central airway resistance at 20 Hz, *R5–R20* peripheral airway resistance as the difference between 5 Hz and 20 Hz, *RF* resonant frequency, *X5* reactance at 5 Hz, *AX* reactance area, *6MWT* 6-min walk test, bpm beats per minute, *SGRQ* St George’s Respiratory Questionnaire

**Table 2:** Within aclidinium: baseline versus post-treatment

Parameter	Baseline	Post-aclidinium	Difference (95 % CI)	*P* value
FEV_1_ (L)	1.21	1.32	0.11 (0.03, 0.18)	0.009
FVC (L)	2.67	2.95	0.28 (0.05, 0.50)	0.02
FEF25–75 (L s^−1^)	0.46	0.48	0.02 (−0.04, 0.08)	0.50
RVC (L)	3.12	3.43	0.30 (0.19, 0.42)	<0.0001
RVC/FVC	1.17	1.18	0.01 (−0.08, 0.10)	0.76
R5 (kPa L^−1^ s)	0.78	0.71	−0.07 (−0.15, 0.01)	0.07
R20 (kPa L^−1^ s)	0.42	0.41	−0.01 (−0.04, 0.03)	0.61
R5–R20 (kPa L^−1^ s)	0.35	0.29	−0.06 (−0.11, −0.01)	0.02
X5 (kPa L^−1^ s)	−0.38	−0.34	0.03 (−0.04, 0.11)	0.36
RF (Hz)	28.54	26.32	−2.22 (−4.37, −0.08)	0.04
AX (kPa L^−1^)	4.29	3.58	−0.71 (−1.49, 0.07)	0.07
6MWT				
Distance (m)	406	442	36 (1, 70)	0.045
Post−walk oxygen saturation (%)	91	92	0 (−2, 2)	0.73
Post-walk heart rate (bpm)	76	79	3 (0, 6)	0.08
Post-walk dyspnoea (Borg scale)	2.7	2.2	−0.6 (−1.2, 0.1)	0.08
Post-walk fatigue (Borg scale)	2.2	1.7	−0.5 (−1.0, 0.0)	0.04
SGRQ				
Symptoms score	45.10	37.76	−7.35 (−14.12, −0.57)	0.04
Total score	36.75	35.78	−0.97 (−4.51, 2.57)	0.56

Mean values are shown

*FEV*_*1*_ forced expiratory volume in 1 s, *FVC* forced vital capacity, *FEF*_*25–75*_ forced mid-expiratory flow between 25 and 75 % of forced vital capacity, *RVC* relaxed vital capacity, *R5* total airway resistance at 5 Hz, *R20* central airway resistance at 20 Hz, *R5–R20* peripheral airway resistance as the difference between 5 Hz and 20 Hz, *RF* resonant frequency, *X5* reactance at 5 Hz, *AX* reactance area, *6MWT* 6-min walk test, *bpm* beats per minute, *SGRQ* St George’s Respiratory Questionnaire

**Table 3:** Within tiotropium: baseline versus post-treatment

Parameter	Baseline	Post-tiotropium	Difference (95 % CI)	*P* value
FEV_1_ (L)	1.20	1.35	0.15 (0.10, 0.20)	<0.0001
FVC (L)	2.73	2.97	0.24 (0.10, 0.39)	0.003
FEF25–75 (L s^−1^)	0.47	0.53	0.06 (0.02, 0.10)	0.004
RVC (L)	3.22	3.44	0.22 (0.04, 0.41)	0.02
RVC/FVC	1.23	1.18	−0.05 (−0.16, 0.06)	0.32
R5 (kPa L^−1^ s)	0.74	0.71	−0.03 (−0.09, 0.03)	0.30
R20 (kPa L^−1^ s)	0.42	0.41	−0.01 (−0.04, 0.02)	0.36
R5–R20 (kPa L^−1^ s)	0.31	0.30	−0.01 (−0.07, 0.04)	0.58
X5 (kPa L^−1^ s)	−0.37	−0.33	0.05 (−0.01, 0.11)	0.12
RF (Hz)	28.90	26.13	−2.77 (−4.58, −0.96)	0.006
AX (kPa L^−1^)	4.21	3.66	−0.55 (−1.16, 0.05)	0.07
6MWT				
Distance (m)	429	437	9 (−13, 30)	0.40
Post-walk oxygen saturation (%)	92	93	0 (−1, 2)	0.64
Post-walk heart rate (bpm)	75	76	1 (−2, 4)	0.49
Post-walk dyspnoea (Borg scale)	2.8	2.1	−0.7 (−1.2, −0.1)	0.03
Post-walk fatigue (Borg scale)	2.4	2.1	−0.3 (−0.8, 0.2)	0.21
SGRQ				
Symptoms score	47.34	40.03	−7.31 (−14.62, 0.00)	0.05
Total score	38.85	36.50	−2.35 (−5.71, 1.02)	0.15

Mean values are shown

*FEV*_*1*_ forced expiratory volume in 1 s, *FVC* forced vital capacity, *FEF*_*25–75*_ forced mid-expiratory flow between 25 and 75 % of forced vital capacity, *RVC* relaxed vital capacity, *R5* total airway resistance at 5 Hz, *R20* central airway resistance at 20 Hz, *R5–R20* peripheral airway resistance as the difference between 5 Hz and 20 Hz, *RF* resonant frequency, *X5* reactance at 5 Hz, *AX* reactance area, *6MWT* 6-min walk test, *bpm* beats per minute, *SGRQ* St George’s Respiratory Questionnaire

**Fig. 5:**
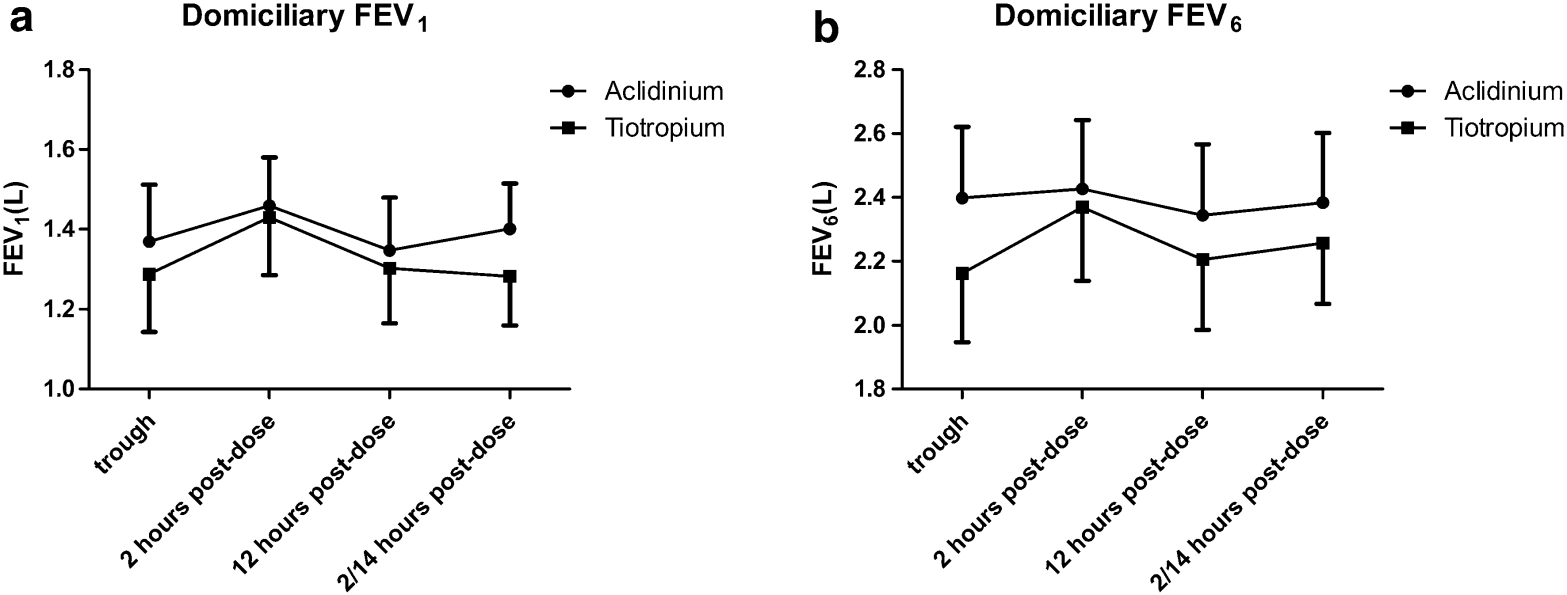
Diurnal profiles with either tiotropium given once daily in the morning or aclidinium given twice daily in the morning and evening, for the last week of each randomized treatment. Data are depicted as means and SEM. **a** *FEV*_*1*_ forced expiratory volume in 1 s **b** *FEV*_*6*_ forced expiratory volume in 6 s. Data are shown for the morning trough measurement (i.e., pre-dose for aclidinium and tiotropium), 2 and 12 h post dose (i.e., trough for aclidinium), and 2 h post the evening dose of aclidinium or 14 h after the morning dose of tiotropium. There were no significant differences between tiotropium and aclidinium at any time points

There were no significant differences between treatments for the 6-min walk test (Table 1). Six-min walk distance and post-walk fatigue significantly improved from baseline with ACL, while post-walk dyspnea improved with TIO (Tables 2, 3). Post-walk heart rate and oxygen saturation were not significantly altered by either treatment (Fig. 5).

SGRQ symptom score significantly improved with both drugs from baseline, but there was no difference between treatments total or symptoms score (Tables 1, 2, 3). Mean BDI was 6.54, while TDI was significantly improved by ACL (1.0) versus TIO (–0.54): mean difference 1.54 (95 % CI 0.39–2.69), *P* = 0.013.

## Discussion

The results of this proof of concept study showed no significant differences between randomized treatments in any IOS or spirometry outcomes measured at trough after chronic dosing with TIO and ACL when used as triple therapy in patients with COPD. We found no significant difference between treatments in the primary outcome of R5, although neither drug produced any significant improvements in R5 from baseline. Within the power constraints of the sample size, we cannot exclude the possibility that we may have missed a difference in R5 smaller than 0.1 kPa L^−1^ s, which we considered to be a clinically important difference. Other IOS outcomes including R20, X5, and AX were not significantly improved from baseline with either drug from baseline. Nonetheless, we showed that both treatments produced comparable significant improvements from baseline in RF, while ACL also produced a significant improvement in R5–R20. However, the clinical relevance of small changes in IOS outcomes in COPD is uncertain as there are currently no published data with regard to minimal important differences. In one cross-sectional study in COPD patients, peripheral airway resistance correlated with FEV_1_ and FEF_25–75_ but not with the Medical Research Council (MRC) dyspnea score [14]. In a baseline cross-sectional analysis of the ECLIPSE study in COPD, IOS was found to be reproducible and was able to define the severity of disease according to global initiative for chronic obstructive lung disease (GOLD) status [15].

We are aware of only one other clinical trial evaluating triple therapy in COPD using IOS. In that study, Williamson et al. enrolled patients with severe COPD (FEV_1_ 42 %) comparing 2 weeks of TIO and placebo as add-on to ICS/LABA [9]. IOS outcomes including R5, R20, and X5 were not significantly different after 2 weeks of treatment despite a significant improvement in FEV_1_. This disconnect between improvements in FEV_1_ but not R5 in response to TIO after 2 weeks is consistent with our current findings. Moreover, the mean R5 at baseline on ICS/LABA prior to TIO reported by Williamson et al. was 0.7 kPa L^−1^ s which is similar to baseline R5 values in our patients. Perhaps performing a further evaluation using a full 24-h profile with serial IOS as an area under the curve (AUC) might be able to detect more subtle differences in airway caliber which we did not observe on a single trough measurement.

Whole-body plethysmography is alternative effort-independent test using a panting maneuver which can measure airway resistance (or its reciprocal as conductance) but is more difficult and time consuming to perform than IOS. Singh et al. reported on a comparison of fluticasone/salmeterol versus fluticasone/salmeterol/TIO for 2 weeks in patients with COPD, where the primary outcome of peak specific airway conductance (as AUC 0–4 h) showed a 27 % significant difference in favor of triple therapy, while for trough FEV_1_, there was a significant mean difference of 110 ml [3]. In our study, we showed significant within treatment improvements in mean trough FEV_1_ amounting to 110 ml with ACL and 150 ml with TIO, as compared to the minimal important difference of 100–140 ml [16] .

For visit-based spirometry outcomes including FEV_1_, FVC, and RVC we observed significant within treatment effects compared to baseline in response to TIO and ACL, but no difference between treatments. Moreover, there were no significant differences between treatments for domiciliary diurnal profiles of FEV_1_ and FEV_6_. In a previous comparison of the same doses of TIO and ACL used as monotherapy, there were no differences between drugs in terms of trough pre-dose FEV_1_ measurements after 2 weeks, although the AUC 12–24 h for the visit-based diurnal FEV_1_ profile was significantly better with ACL compared to TIO after chronic dosing [11]. This observed difference in the FEV_1_ AUC 12–24 h corresponds to the 12-h period after taking the evening dose of ACL when the effect of TIO is beginning to wane throughout the night time prior to the next morning dose. Perhaps on reflection, we might have also shown such differences in domiciliary FEV_1_ if we had performed diurnal evening measurements between the 14- and 24-h time point for TIO, although we considered that this was not practical for elderly patients to perform at home. Another possibility is that in the present study, there may be less room for further improvement conferred by LAMA when given as add-on to ICS/LABA compared to its use as monotherapy. In a previous comparison of TIO and GLYC as add-on to ICS/LABA, the mean difference in the primary outcome of trough FEV_1_ amounted to 7 ml [10], as compared to a 40 ml difference between TIO and ACL in the present study.

We used dry powder formulations of TIO and ACL which emit coarse particles >2 µm, which could also explain the somewhat limited improvements in pulmonary function. In this regard, we are not aware of any head to head lung deposition studies comparing TIO and ACL dry powder formulations in COPD. Perhaps using smaller particle formulations such as the fine-mist TIO Respimat inhaler might result in greater improvements in regional lung deposition.

With the development of single inhaler triple therapy, it will be important to know what the impact is on more clinically relevant outcomes. In a retrospective cohort study of 2853 patients with moderate to severe COPD followed up over 4.65 years, there were 996 receiving ICS/LABA (FEV_1_ of 63 %) and 1857 receiving ICS/LABA/LAMA (FEV_1_ of 51 %) [17]. Comparing triple versus dual therapy, there was a 15 % reduction in hospital admissions, 29 % fewer oral corticosteroid bursts, and 26 % lower all-cause mortality, while triple therapy was associated with a fall in serial FEV_1_ of 30 ml over 4 years.

Aside from pulmonary function outcomes, it is also important to consider the impact of treatment upon functional status (6-min walk distance and dyspnoea index) and health-related quality of life (SGRQ) [16]. While we observed no difference between treatments in 6-min walking distance, there was a 36 m mean improvement from baseline with ACL, which exceeds the minimal important difference of 25 m [18, 19]. There was a significant improvement post-walk fatigue with ACL and in post-walk dyspnoea with TIO, but no differences were seen in either outcome between treatments. Furthermore post-walk heart rate and oxygen saturation did not change with either treatment. In terms of quality of life, although the SGRQ symptoms score was significantly improved with both drugs, there was no difference between them. The transition dyspnoea index was significantly improved by ACL versus TIO by a mean difference of 1.54 which exceeded the minimal important difference of 1.0 [20]. The relevance of this finding in the absence of any differences in objective pulmonary function outcomes is uncertain.

In summary, we observed comparable bronchodilator efficacy at trough with TIO and ACL when used as triple therapy in COPD, while IOS was no more sensitive than spirometry. The utility of IOS measurements in COPD requires further evaluation perhaps using a diurnal IOS profile to compare with spirometry or whole-body plethysmography.
